# Manifestations of Abrasion

**Published:** 1897-04

**Authors:** C. F. Piper


### Manifestations of Abrasion.

BY C. F. PIPER.

Perhaps at no time during the life and existence of the teeth in the mouth are they entirely free from all destructive agents.

When in use, normally, in mastication, friction from antagonizing teeth and from solid particles in the food, slowly but surely, wears them away. Imperfection, and irregularities on the tooth-surface, may form lodgment for vitiated mucous, which, becoming acid, may lead to dissolution of their surfaces, or systemic disorders may give rise to acid saliva which gradually, but certainly, produces waste.

Whether these various processes are entirely distinct, allowing us to clearly classify them, as to cause and effect, or whether each is, in some way, dependent upon the other, is not fully known.

Some would make different classifications of tooth-waste, using the term *attrition* for the wearing away of tooth-substance by mastication. *Erosion*, where there is gradual disappearance of tooth-substance, leaving a smooth, polished surface, the cause being obscure, and *abrasion* for those destructions of tooth-substance through friction with foreign bodies, such as clasps, tooth-brush, pipe, etc.

As there is no special boundary between these different causes each seemingly overlapping and aiding the other, we are, perhaps, justified in using them all under the one head of "Abrasion," and dividing this into two classes—Mechanical and Chemical. The former being destructive, due to mechanical forces, the latter removal of lime-salts by chemical solution.

Even these two classes are not distinct, for the smooth, polished surfaces left by chemical abrasion must be due, somewhat, to friction between lips, tongue and food. Both occur in persons o robust as well as delicate constitutions.

In both cases, if much waste has occurred, there is an effort made to protect the pulp by a secondary deposit of lime in the dental tubuli.

Again, in either case there is no change in color so long as the action is in the enamel, but the dentine being reached there is a change of color, due to solidifying of the dental tubules.

Mechanical abrasion is a term applied to those lesions of tooth-substance arising from friction-wear, and is entirely mechanical. It occurs at all periods of life, being occasionally found on the temporary teeth, but most marked in teeth of adults, and especially of old people, giving rise to the so-called double teeth.

The character of this abrasion depends on the position of the teeth in the upper and lower jaw, and as their variations are many the varieties of abrasion are, likewise, very numerous. Often the teeth stand so as to receive the friction on their sides, thus causing a wearing away at that point. When the friction is caused by the use of a tooth-brush, the cuspids are the ones most attacked, due to their great prominence in the dental-arch. Persons addicted to the smoking of clay pipes have marked forms of abrasion, leading to almost entire destruction of cuspids and bicuspids.

It has been known, according to some writers, to occur upon the proximal surfaces of teeth which came in contact with adjoining teeth, and is produced by the motion of these teeth within the sockets during mastication.

Chemical abrasion is the wearing away of tooth-substance, due to a solvent material, claimed by some to be lactic acid, and by others uric acid. It is rarely found in youth, being most common after 25 years of age.

It usually begins near an accumulation of mucous. When found any distance from the gum-border, the action is attributed to secretions from the mucous-glands of the tongue and lips; they, generally, being much irritated about the abraded region. Some claim this action is limited to the anterior part of the incisors and cuspids, but this is not the case, as it is found on all teeth and on all sides of them.

The most common manifestation is found on the incisors in the form of grooves, running near the gingival border, or from the border of the gum to the cutting edge. Sometimes they may run transversely across the tooth, appearing as though a sharp fine fibre had been drawn across it.

A characteristic feature of all these grooves is, that they always present a highly-polished and glassy surface.

